# Cholinergic deficiency in Parkinson’s disease patients with visual hallucinations

**DOI:** 10.1093/brain/awae186

**Published:** 2024-06-12

**Authors:** Emile d’Angremont, Sygrid van der Zee, Sofie Slingerland, Anne C Slomp, Erik F J de Vries, Teus van Laar, Iris E Sommer

**Affiliations:** Department of Biomedical Sciences, University Medical Center Groningen, University of Groningen, 9713 GZ Groningen, The Netherlands; Department of Neurology, University Medical Center Groningen, University of Groningen, 9713 GZ Groningen, The Netherlands; Department of Neurology, University Medical Center Groningen, University of Groningen, 9713 GZ Groningen, The Netherlands; Department of Neurology, University Medical Center Groningen, University of Groningen, 9713 GZ Groningen, The Netherlands; Department of Nuclear Medicine and Molecular Imaging, University Medical Center Groningen, University of Groningen, 9713 GZ Groningen, The Netherlands; Department of Neurology, University Medical Center Groningen, University of Groningen, 9713 GZ Groningen, The Netherlands; Department of Biomedical Sciences, University Medical Center Groningen, University of Groningen, 9713 GZ Groningen, The Netherlands

**Keywords:** Parkinson’s disease, visual hallucinations, acetylcholine, (2*R*,3*R*)-5-(2-[^18^F]fluoroethoxy)benzovesamicol PET

## Abstract

Visual hallucinations can increase the burden of disease for both patients with Parkinson’s disease and their caregivers. Multiple neurotransmitters have been implicated in the neuropathology of visual hallucinations, which provide targets for treatment and prevention. In this study, we assessed the association between cholinergic denervation and visual hallucinations in Parkinson’s disease *in vivo*, using PET imaging of the cholinergic system.

A total of 38 patients with Parkinson’s disease participated in this study. A group of 10 healthy subjects, matched for age, sex and education, was included for comparison. None of the participants used cholinergic drugs. Thirteen patients who had experienced visual hallucinations in the past month (VH+) were compared with 20 patients who had never experienced visual hallucinations in their lives (VH−). Cholinergic system integrity was assessed with PET imaging using ^18^F-fluoroethoxybenzovesamicol as the tracer. We assessed the differences in tracer uptake between groups by cluster-based analysis and by analysis of predefined regions of interest consisting of the ventral visual stream, the dorsal attentional network, the ventral attentional network and the lateral geniculate nucleus and mediodorsal nucleus of the thalamus.

The Parkinson’s disease group (*n* = 38) showed an extensive pattern of decreased tracer uptake throughout the brain compared with the controls (*n* = 10). Within the Parkinson’s disease group, the VH+ group (*n* = 13) showed a cluster of decreased tracer uptake compared with the VH− group (*n* = 20), which covered most of the left ventral visual stream and extended towards superior temporal areas. These results were mirrored in the regions of interest-based analysis, in which the VH+ group showed the strongest deficits in the left inferior temporal gyrus and the left superior temporal gyrus compared with the VH− group.

Visual hallucinations in Parkinson’s disease are associated with a marked cholinergic deficiency in the left ventral visual stream and the left superior temporal lobe, in addition to an extensive global cholinergic denervation in the general Parkinson’s disease population.

## Teaser

d'Angremont *et al*. use PET imaging of the cholinergic system to study the relationship between cholinergic denervation and visual hallucinations in Parkinson’s disease. They find greater denervation in brain areas related to visual processing in patients with visual hallucinations compared to patients without this symptom.

## Introduction

Visual hallucinations (VH) are the most common psychotic symptom in Parkinson’s disease (PD), affecting ∼25% of the PD population, with an increasing prevalence in later stages of the disease.^1^ VH in PD are often complex, taking the form of persons or animals.^2^ Psychotic symptoms can significantly impact the quality of life of patients and increase caregiver burden.^3^ Moreover, presence of VH is associated with increased risk for nursing home placement and higher mortality.^4,5^

Several cognitive models for VH in PD exist. Collerton *et al*.^6^ recently proposed an integrated VH framework, consistent with the existing models of complex VH, and highlighted its implications for further research. Arguably the most prominent cognitive model is the perception and attention deficit (PAD) model. This model states that poor visual perception in the ventral visual stream leads to a perceived image that is biased towards expectancies rather than sensory input.^6^ Imprecise data comparison owing to impaired object attention in turn makes the hallucination fail to be updated to a more veridical image. The PAD model also poses that a cortical cholinergic hypoactivity lies at the basis of these deficits.^7^ Many of the other cognitive VH models (particularly the attentional networks model by Shine *et al*.,^8^ the activation input modulation model by Diederich *et al*.^9^ and, to a lesser extent, the thalamocortical dysrhythmia model by Onofrj *et al*.^10^) show overlap with the PAD model in terms of brain systems involved and relevant cholinergic pathology.

VH were long considered as a side-effect of dopaminergic treatment, but now it has become clear that dopaminergic stimulation cannot fully explain the development of VH in PD.^11^ Both neurochemical and structural neuroimaging studies have hinted at a deficiency in central cholinergic activity as an underlying component for the development of VH.^12-15^ However, many of these studies have been performed post-mortem or with an indirect measure of cholinergic system integrity, such as voxel-based morphometry and structural connectivity. Therefore, the exact association between cortical cholinergic activity *in vivo* and the presence of VH in PD is currently not well understood.

In the present study, we investigated cholinergic differences between PD patients and matched controls, and also between patients with and without VH, using the PET tracer (2R,3R)-5-(2-[^18^F]fluoroethoxy)benzovesamicol (^18^F-FEOBV), which binds to the vesicular acetylcholine transporter (VAChT). For the latter comparison, we compared patients who experienced hallucinations in the past month with those who never experienced any VH. This is the first study to apply ^18^F-FEOBV PET in PD patients with and without VH. We hypothesized that PD patients with VH will show a more advanced cholinergic denervation than patients without VH, especially in areas related to visual processing and attentional networks, as suggested by the PAD model.

## Materials and methods

### Subjects

For this study, we collected data of 38 PD patients and 10 healthy controls, who were initially recruited in the context of a study assessing the relationship between ^18^F-FEOBV PET and short-latency afferent inhibition, a potential cholinergic biomarker.^16^ All subjects underwent ^18^F-FEOBV PET imaging and a T1-weighted MRI scan, in addition to standardized assessments on psychotic symptoms and cognition (see later). Subjects using cholinesterase inhibitors or drugs with anticholinergic effects (e.g. clozapine) were excluded.^17,18^ The healthy controls were matched group-wise to the patient group based on sex, age and education. Additional exclusion criteria for the healthy control subjects included the presence of a neurological or neurodegenerative disease. A full list of the inclusion and exclusion criteria can be found in the Supplementary material. All subjects had provided written informed consent in accordance with the Declaration of Helsinki, and the study was conducted according to the Good Clinical Practice guidelines. The study was approved by the ethical review board of the University Medical Center Groningen.

Thirteen patients were selected from the overall PD group, who reported having experienced VH in the past month (the VH+ group). Twenty patients had never experienced visual hallucinations and were used as a control group (the VH− group). The remaining five patients, who had experienced VH but not in the past month, were excluded from the VH-based analysis.

### Clinical assessment

The presence, frequency and severity of psychotic symptoms were assessed with the Questionnaire for Psychotic Experiences (QPE), administered as a structured interview, also including the partner of the patient when present.^19^ Global cognition was assessed using the Montreal Cognitive Assessment (MoCA),^20^ and visuospatial cognition was assessed with the Map Search from the Test of Everyday Attention^21^ and the judgment of line orientation.^22^ Motor performance was assessed with the Movement Disorder Society Unified Parkinson’s Disease Rating Scale part 3 (MDS-UPDRS-III).^23^

### Image acquisition

All participants were injected with ∼200 MBq of ^18^F-FEOBV, with a range of 180–220 MBq. At 210 min after injection, participants underwent a low-dose CT for attenuation and scatter correction, and a PET scan of the brain. Scans were acquired with a Siemens Biograph mCT scanner. PET data were acquired in six 5-min frames to allow for motion correction. For all participants, anatomical three-dimensional T1-weighted magnetic resonannce images with 1 mm × 1 mm × 1.2 mm resolution acquisition (repetition time = 2300 ms, echo time = 2.98 ms, flip angle = 9°) were obtained on a Siemens 3 T machine.

### Image processing

Image preprocessing was performed using the Statistical Parametric Mapping software package (SPM v.12, Wellcome Trust Center for Neuroimaging). All PET frames were corrected for motion to the first frame for each subject using a rigid body spatial transformation, after which the frames were averaged. The averaged PET images were co-registered with the individual T1-weighted MRI scans and intensity normalized to a reference region, resulting in parametric standardized uptake value ratio (SUVr) images. As the reference region, we used an eroded version of the supratentorial white matter mask, resulting from a FreeSurfer (http://surfer.nmr.mgh.harvard.edu/) segmentation of the MRI scans, as described previously.^24^ We corrected the parametric SUVr images for partial volume effects with the Müller-Gärtner method,^25^ using the PETPVE12 toolbox in SPM,^26^ assuming CSF to have zero intensity. A grey and white matter segmentation using the CAT12 toolbox with default settings was used for this purpose. Partial volume effect correction was applied to ensure that our results are not merely based on a difference in brain atrophy between groups.^27^ Finally, the images were spatially normalized to the Montreal Neurological Institute (MNI) standard space, using the transformation matrix resulting from the CAT12 segmentation, and smoothed using an 8 mm × 8 mm × 8 mm kernel.

### Statistical analysis

We performed a whole-brain cluster-based analysis on the normalized and smoothed parametric SUVr images, controlling for age, sex and disease duration (for patient groups), using standard SPM statistical methods. We set the primary threshold for statistical difference between groups at the voxel level at *P* < 0.001 and chose the cluster extend threshold in order to correct for family-wise error with *P* < 0.05, based on a random field theory approach implemented in SPM. We assessed three different contrasts for the cluster-wise analysis. First, we compared healthy controls (*n* = 10) with PD patients (*n* = 38). Second, we compared the VH+ (*n* = 13) and VH− (*n* = 20) subgroups with each other. Third, we compared the VH+ (*n* = 13) and the VH− (*n* = 20) groups with the healthy controls (*n* = 10) (results of the third comparison are presented in Supplementary material).

In addition to the cluster-based analysis, we compared the VH+ and VH− groups with each other based on predefined regions of interest (ROIs). The ROI selection was based on the PAD model,^7^ including areas within the ventral visual pathway (VIS), the ventral attentional network (VAN), the dorsal attentional network (DAN) and the lateral geniculate nucleus and mediodorsal nucleus of the thalamus (a list of ROIs can be found in Supplementary Table 1). The mediodorsal nucleus of the thalamus was chosen because it receives direct cholinergic projections from the basal forebrain^28^ and is involved in the PAD model as initially proposed by Collerton *et al*.^7^ Definition of the ROIs included in the VIS, VAN and DAN was based on the functional areas identified by Power *et al*.^29^ and applied to the neuromorphometrics brain atlas in MNI space. We extracted the mean SUVr values per ROI using SPM. Further statistical analysis was performed in R v.4.2.2,^30^ where we initially standardized all values per ROI to a mean of zero and SD of one over all subjects included in the VH-related analysis, then applied a linear regression model for all ROIs, with VH state as an independent variable and with age, sex and disease duration as covariates. We used a false discovery rate correction per ROI group.

For both the cluster-based analysis and the ROI-based analysis, we performed additional statistical tests with the MoCA overall score as an additional covariate.

## Results

### Participants

Table 1 shows an overview of the demographics of all participants. The PD group had a significantly lower score on the Map Search compared with the healthy controls (*P* < 0.001), but there were no differences in age, sex and educational level. The VH+ and VH− subgroups were also comparable regarding age, sex, education and disease duration. However, the VH+ group had a significantly more advanced disease stage based on the Hoehn and Yahr scale, compared with the VH− group (*P* = 0.033). There were no significant differences between patients with and without VH in MoCA score (*P* = 0.083), Map Search (*P* = 0.060) and judgment of line orientation (*P* = 0.317), nor in levodopa-equivalent daily dose (*P* = 0.748) or the percentage of dopamine agonist users (*P* = 0.651). All patients in the VH+ group reported having complex VH, which also included the perception of bugs (*n* = 2), smoke (*n* = 1) and people passing by the window (*n* = 1). Eight patients reported experiencing minor hallucinatory phenomena, such as visual illusions and feeling of a presence, additional to complex VH. One patient in the VH+ group suffered from paranoid delusion. Four patients in the VH+ group and two patients in the VH− group had a clinical diagnosis of PD-related dementia. Only two patients had a MoCA score of <21, both in the VH− group.

**Table 1:** Demographic data and clinical scores of patients and controls and of the visual hallucination subgroups

Parameter	Controls (*n* = 10)	Parkinson’s disease (*n* = 38)	*P*- value	VH− (*n* = 20)	VH+ (*n* = 13)	*P*-value
Age, years	67.6 (8.4)	67.5 (8.4)	0.981	66.0 (8.8)	69.5 (7.5)	0.245
Sex, percentage male	80	79	1	75	84.6	0.822
ISCED, median [IQR]	6 [3–6]	3 [3–6]	0.158	3 [3–6]	3 [3–6]	0.518
MoCA	26.5 (2.1)	24.8 (3.7)	0.077	26.2 (3.2)	24.5 (2.2)	0.083
Map Search	58.1 (6.9)	40.3 (16.9)	**<0.001**	46.5 (14.8)	34.5 (18.2)	0.060
Judgment of line orientation	25.9 (4.2)	25.3 (4.9)	0.716	26.7 (4.3)	25.2 (4.0)	0.317
MDS-UPDRS-III	–	–	–	25.6 (10.3)	30.1 (9.6)	0.211
Hoehn and Yahr, median (range)	–	–	–	2 (1–3)	2 (2–4)	**0**.**033**
Levodopa equivalent daily dose, mg	–	–	–	977 (450)	1058 (816)	0.748
Dopamine agonist use (percentage yes)	–	–	–	55	69	0.651
Disease duration, years, median [IQR]	–	–	–	4 [3–6.3]	5 [3–7]	0.853
MDS-UPDRS-III asymmetry index	–	–	–	0.26 (0.7)	−0.01 (0.4)	0.175

Values are given as the mean (SD) unless indicated otherwise. Values of *P* < 0.05 are in bold. IQR = interquartile range; ISCED = International Standard Classification of Education; MDS-UPDRS-III = Movement Disorder Society Unified Parkinson’s Disease Rating Scale part 3; MoCA = Montreal Cognitive Assessment; VH− = Parkinson’s disease without visual hallucinations; VH+ = Parkinson’s disease with visual hallucinations.

### Cluster-based analysis

#### Parkinson’s disease versus healthy controls

**Figure 1:**
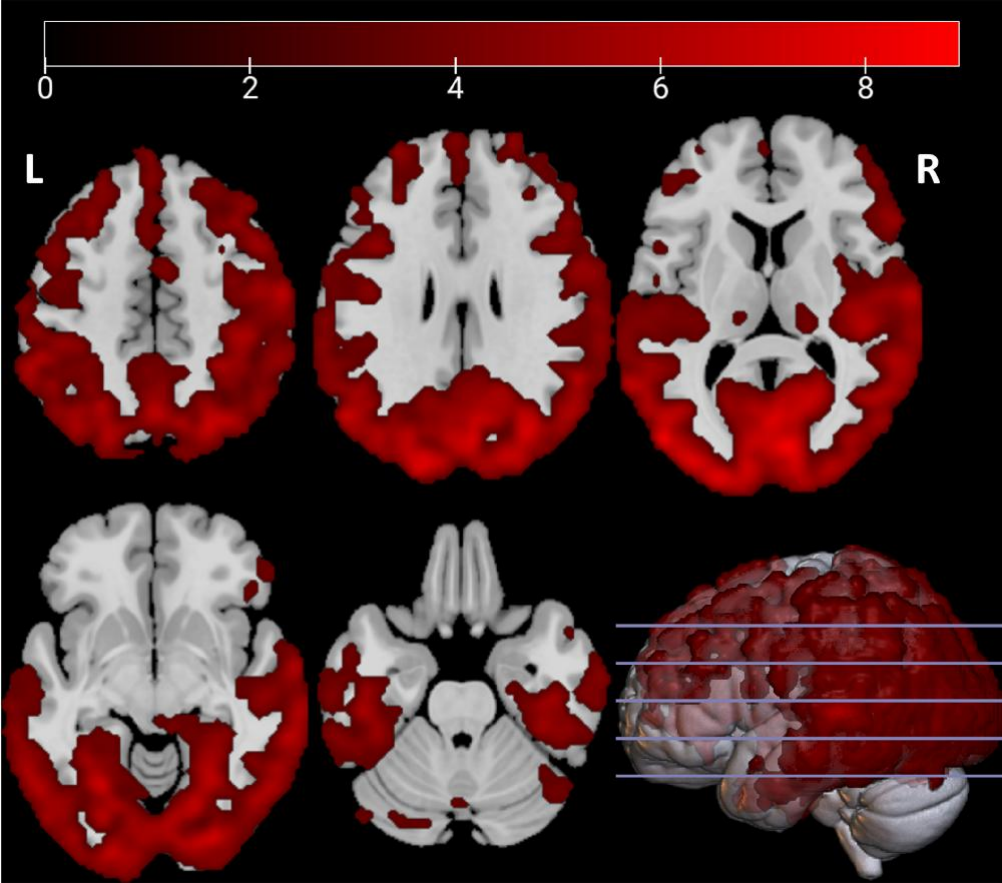
**Decreased tracer uptake in patients (*n* = 38) compared with healthy controls (*n* = 10).** Depicted are *t*-values, corrected for age and sex. R = right.

We found an extensive cluster (85 184 voxels, *P* < 0.0001) of lower tracer uptake in the PD group compared with healthy controls (Fig. 1). The cluster exhibited the strongest difference in the right calcarine cortex, but included the whole occipital lobe and extended towards both left and right parietal, temporal and lateral frontal areas. A separate, smaller cluster (2050 voxels, *P* < 0.0001) was found in the left frontal pole and the medial frontal gyrus. No regions were observed with higher tracer uptake in PD patients versus controls.

#### Visual hallucinations versus no visual hallucinations

**Figure 2:**
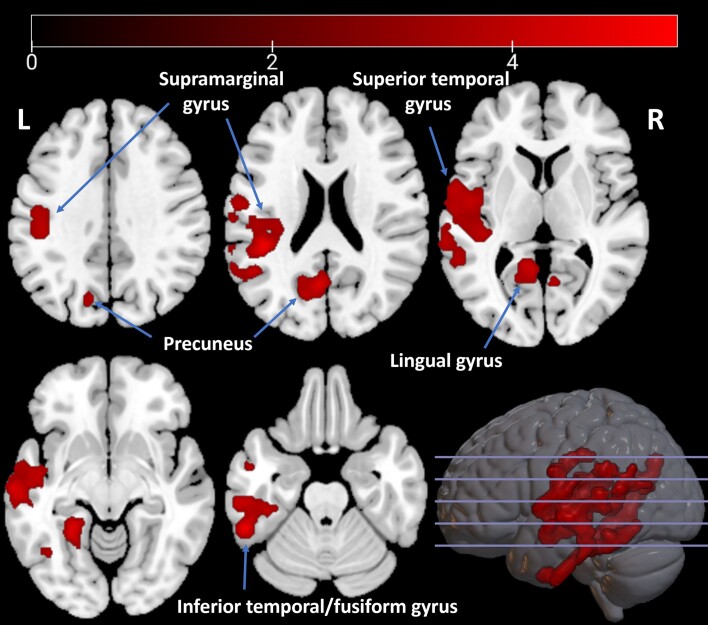
**Decreased tracer uptake in patients with visual hallucinations (*n* = 13) compared with patients without visual hallucinations (*n* = 20).** Depicted are *t*-values, corrected for age, sex and disease duration. L = left; R = right.

In direct comparison between the VH+ and VH− groups, we found one cluster of significantly lower uptake in the VH+ group (6625 voxels, *P* < 0.0001) that showed the largest difference in the left precuneus and that extended through the left lingual gyrus and the left fusiform gyrus towards the left inferior temporal gyrus and from there towards the middle and superior temporal gyri and the supramarginal gyrus (Fig. 2).

**Figure 3:**
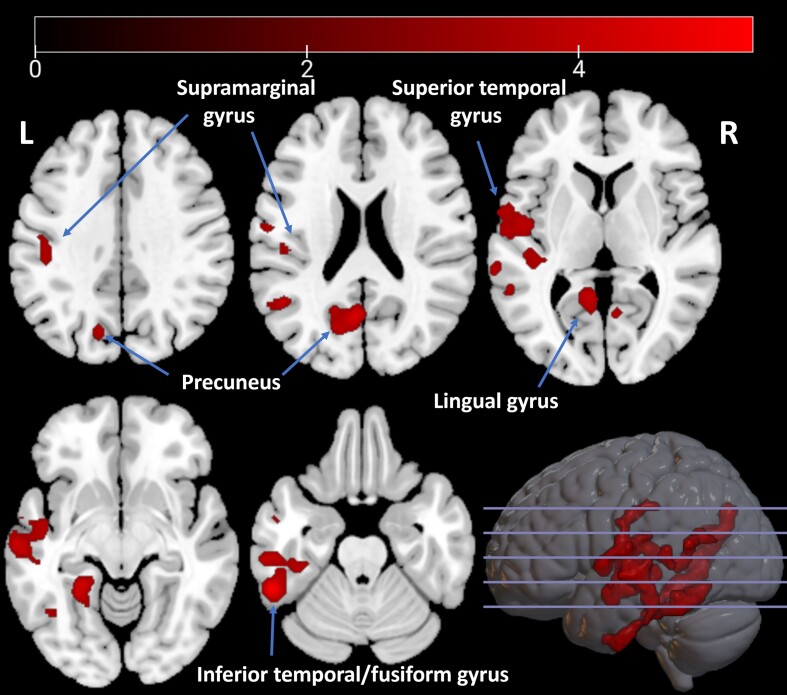
**Decreased tracer uptake in patients with visual hallucinations (*n* = 13) compared with patients without visual hallucinations (*n* = 20).** Depicted are *t*-values, corrected for age, sex, disease duration and Montreal Cognitive Assessment score. L = left; R = right.

With the addition of MoCA as a covariate, the cluster reduced in size (3443 voxels, *P* < 0.0001) and no longer included the left supramarginal gyrus, but all other areas remained included (Fig. 3).

We did not find regions with higher ^18^F-FEOBV binding in the VH+ group compared with the VH− group.

The regions with lower-than-normal tracer uptake in the VH− group, in comparison to controls, were largely limited to posterior brain areas, especially in the left hemisphere, whereas in the VH+ group these regions extended towards the anterior temporal lobe and included more frontal regions (Supplementary Fig. 1).

### Regions of interest-based analysis

#### Visual hallucinations versus no visual hallucinations

We observed decreased tracer uptake in the VH+ group compared with the VH− group in all included ROIs of the ventral visual stream in the left hemisphere, except for the superior occipital gyrus (Table 2). In the right hemisphere, only the lingual gyrus and inferior temporal gyrus showed decreased tracer uptake. All these ROIs of the ventral visual stream remained significantly different with the addition of MoCA as a covariate (Table 3). In the VAN, the left superior temporal gyrus, the planum temporale and the triangular part of the inferior frontal gyrus showed significant differences, but the last did not remain significant after adding MoCA as covariate. No significant differences were found in the right hemisphere after correction for multiple comparisons in this network. A similar pattern emerged for the DAN, where all included ROIs except for the superior parietal lobe had significantly lower uptake in the VH+ group in the left hemisphere. These significant differences in the DAN disappeared after adding MoCA as a covariate. No significant differences were found in the thalamic subregions. Overall, the effect was strongest in the left inferior temporal gyrus (β* = −1.00), closely followed by the left superior temporal gyrus (β* = −0.99), when correcting for MoCA (Table 3).

**Table 2:** Results of region of interest-based analysis between patients with visual hallucinations (*n* = 13) and without visual hallucinations (*n* = 20), corrected for age, sex and disease duration

Region of interest	Left			Right		
	Effect size	*P*-value	Corrected *P*-value	Effect size	*P*-value	Corrected *P*-value
**Ventral visual stream**						
Superior occipital gyrus	−0.61	0.062	0.076	−0.23	0.539	0.539
Middle occipital gyrus	−0.81	**0**.**015**	**0**.**027**	−0.35	0.349	0.369
Inferior occipital gyrus	−0.79	**0**.**015**	**0**.**027**	−0.48	0.192	0.216
Cuneus	−0.82	**0**.**012**	**0**.**027**	−0.62	0.063	0.076
Calcarine cortex	−0.85	**0**.**009**	**0**.**027**	−0.68	**0**.**039**	0.059
Lingual gyrus	−0.84	**0**.**004**	**0**.**022**	−0.72	**0**.**012**	**0**.**027**
Occipital fusiform gyrus	−0.79	**0**.**015**	**0**.**027**	−0.62	0.056	0.076
Fusiform gyrus	−0.85	**0**.**003**	**0**.**022**	−0.55	**0**.**035**	0.057
Inferior temporal gyrus	−1.04	**0**.**000**	**0**.**006**	−0.78	**0**.**009**	**0**.**027**
**Ventral attentional network**						
Superior frontal gyrus	−0.67	0.059	0.073	−0.41	0.244	0.244
Inferior frontal gyrus, triangular part	−0.84	**0**.**010**	**0**.**034**	−0.49	0.116	0.129
Frontal operculum	−0.65	**0**.**038**	0.068	−0.61	**0**.**047**	0.068
Planum temporale	−0.96	**0**.**003**	**0**.**015**	−0.75	**0**.**021**	0.054
Superior temporal gyrus	−1.06	**0**.**001**	**0**.**007**	−0.67	**0**.**044**	0.068
**Dorsal attentional network**						
Middle frontal gyrus	−0.79	**0**.**020**	**0**.**048**	−0.44	0.209	0.278
Precentral gyrus	−0.87	**0**.**012**	**0**.**045**	−0.41	0.239	0.287
Superior parietal lobe	−0.62	0.084	0.126	−0.31	0.414	0.414
Middle temporal gyrus	−0.98	**0**.**002**	**0**.**023**	−0.71	**0**.**036**	0.072
Angular gyrus	−0.81	**0**.**015**	**0**.**045**	−0.38	0.323	0.352
Precuneus	−0.91	**0**.**008**	**0**.**045**	−0.69	**0**.**048**	0.082
**Thalamic subregion**						
Lateral geniculate nucleus	−0.39	0.196	0.393	−0.59	0.071	0.285
Mediodorsal nucleus	−0.25	0.429	0.485	−0.22	0.485	0.485

Negative effect sizes depict lower uptake in the patient group with visual hallucinations. *P*-values were corrected for false discovery rate per region of interest group. Values of *P* < 0.05 are in bold.

**Table 3:** Results of region of interest-based analysis between patients with visual hallucinations (*n* = 13) and without visual hallucinations (*n* = 20), corrected for age, sex, disease duration and Montreal Cognitive Assessment score

Region of interest	Left			Right		
	Effect size	*P*-value	Corrected *P*-value	Effect size	*P*-value	Corrected *P*-value
**Ventral visual stream**						
Superior occipital gyrus	−0.66	0.054	0.082	−0.23	0.543	0.543
Middle occipital gyrus	−0.79	**0**.**022**	**0**.**048**	−0.37	0.345	0.365
Inferior occipital gyrus	−0.75	**0**.**025**	**0**.**048**	−0.48	0.208	0.234
Cuneus	−0.82	**0**.**016**	**0**.**048**	−0.62	0.076	0.091
Calcarine cortex	−0.82	**0**.**015**	**0**.**048**	−0.64	0.060	0.084
Lingual gyrus	−0.78	**0**.**008**	**0**.**048**	−0.70	**0**.**019**	**0**.**048**
Occipital fusiform gyrus	−0.74	**0**.**026**	**0**.**048**	−0.62	0.068	0.087
Fusiform gyrus	−0.80	**0**.**007**	**0**.**048**	−0.52	0.055	0.082
Inferior temporal gyrus	−1.00	**0**.**001**	**0**.**014**	−0.74	**0**.**016**	**0**.**048**
**Ventral attentional network**						
Superior frontal gyrus	−0.55	0.120	0.150	−0.29	0.404	0.404
Inferior frontal gyrus, triangular part	−0.69	**0**.**025**	0.085	−0.41	0.197	0.219
Frontal operculum	−0.53	0.084	0.139	−0.50	0.099	0.142
Planum temporale	−0.86	**0**.**007**	**0**.**037**	−0.68	**0**.**041**	0.103
Superior temporal gyrus	−0.99	**0**.**002**	**0**.**017**	−0.60	0.079	0.139
**Dorsal attentional network**						
Middle frontal gyrus	−0.64	**0**.**048**	0.116	−0.31	0.371	0.435
Precentral gyrus	−0.72	**0**.**028**	0.085	−0.29	0.399	0.435
Superior parietal lobe	−0.54	0.142	0.213	−0.23	0.549	0.549
Middle temporal gyrus	−0.90	**0**.**005**	0.058	−0.64	0.064	0.127
Angular gyrus	−0.76	**0**.**026**	0.085	−0.34	0.389	0.435
Precuneus	−0.84	**0**.**015**	0.085	−0.63	0.079	0.135
**Thalamic subregion**						
Lateral geniculate nucleus	−0.35	0.272	0.544	−0.45	0.153	0.544
Mediodorsal nucleus	−0.14	0.664	0.721	−0.11	0.721	0.721

Negative effect sizes depict lower uptake in the patient group with visual hallucinations. *P*-values were corrected for false discovery rate per region of interest group. Values of *P* < 0.05 are in bold.

#### *Post hoc* analysis

Given the differences in results between the left and right hemisphere, we performed a *post hoc* analysis in which we correlated asymmetry in tracer uptake with asymmetry in the MDS-UPDRS-III, similar to Horsager *et al*.^31^ As exemplar, we chose the ROI with the largest effect size in both hemispheres, i.e. the inferior temporal gyrus. In line with the findings of Horsager *et al*.,^31^ we observed a significant correlation between both asymmetry indices (*r* = 0.42, *P* = 0.015; Supplementary Fig. 2). The summary statistics of the MDS-UPDRS-III asymmetry index are shown in Table 1.

## Discussion

We found reduced VAChT expression in PD patients compared with controls in extensive areas of the brain, including all major lobes of the neocortex, using ^18^F-FEOBV PET imaging. The global cholinergic deficiency observed in PD compared with matched controls is in line with previous findings.^31-33^ However, our data show, for the first time, that patients with recent VH have a more severe reduction in cortical VAChT expression compared with those without VH, predominantly in the left ventral visual stream and the superior temporal lobe.

Our study supports the PAD model for the development of VH, because our results show that a cholinergic deficiency in the VH+ group is present in areas related to visual perception, especially belonging to the ventral visual stream. These findings remain significant after correction for MoCA score, suggesting that the difference in cholinergic degeneration cannot be explained fully by a concomitant cognitive deterioration. However, owing to the intricate relationship between cognitive functioning and VH, it is likely that this analysis partly obscured the effect of interest.

An unexpected finding in our study was that the difference between the VH+ and the VH− groups was found predominantly in the left hemisphere. Some previous studies also suggested a stronger involvement of the left hemisphere in the development of psychotic symptoms in patients with PD,^34-36^ but others found a stronger right hemisphere involvement^37,38^ or indicated bilateral dysfunction.^39,40^ Interestingly, asymmetry in tracer uptake was correlated with asymmetry in motor scores, as has been found previously.^31^ Horsager *et al*.^31^ argued that this finding provides evidence for asymmetrical Lewy body pathology. If this is true, the asymmetry in cholinergic denervation found in the present study might reflect a stronger Lewy body pathology in the left hemisphere of the VH+ group compared with the left hemisphere of the VH− group.

A previous functional MRI study from our group, comparing PD patients with VH versus non-hallucinating PD patients, showed reduced activation of the lateral occipital and ventral temporal cortices,^41^ which might reflect the loss of cortical cholinergic innervation found in these areas in the present study. Other studies reported decreased metabolic rates in occipitotemporoparietal regions^35^ and grey matter volume loss in occipital, occipitotemporal, inferior parietal and medial frontal areas.^42^ These findings might also reflect, at least in part, the loss of local cholinergic input in these regions. A review on this topic concluded that most functional imaging studies demonstrated the involvement of impaired visual pathways in the manifestation of VH in PD, although structural imaging studies were less consistent.^43^ Nevertheless, one study investigating the integrity of white matter tracts originating from the nucleus basalis of Meynert, the major source of cholinergic projections to the neocortex, found impaired projections to the parietal and occipital brain areas in patients with VH.^15^ A recent post-mortem study found reduced acetylcholinesterase concentrations in Brodmann’s areas 18 and 19 in PD patients, most prominently in those with VH,^44^ which aligns with the findings in the present study using *in vivo* VAChT imaging. A study assessing the cholinergic-dependent somatosensory inhibition has also reported differences between patients with and without VH,^45^ although we were not able to validate these findings in a recent study applying the same methodology.^16^

Our findings have clinical implications, because a deficiency of cortical cholinergic innervation could, theoretically, be supplemented by cholinomimetics, such as cholinesterase inhibitors. Cholinesterase inhibitors are indicated for PD-related dementia, to improve attentional and executive performance. For bothersome VH, atypical antipsychotics are currently the only available treatment option according to National Institute for Health and Care Excellence guidelines.^46^ However, atypical antipsychotics can have severe adverse effects and can worsen motor features in PD; therefore, alternative treatment options for these symptoms are warranted. A recent meta-analysis from our group confirmed cholinesterase inhibitors to be effective for treatment of hallucinations and delusions in PD, albeit with small effect sizes.^47^ These small effect sizes might be explained, in part, by the multifactorial aetiology of VH, also including dopaminergic and serotonergic stimulation, but it should also be noted that a trial with VH as the primary outcome has rarely been performed. The results from the present study provide the neurochemical basis to substantiate the use of cholinesterase inhibitors in patients with VH, because they have the potency to supplement the cholinergic deficits observed in the VH group.

### Strengths and limitations

^18^F-FEOBV is a reliable presynaptic tracer to assess the distribution of cholinergic nerve terminals *in vivo* in the human brain.^33,48^ This is the first study using this tracer to assess the relationship between cortical cholinergic deficiencies and the presence of VH in PD in a relatively large sample size.

The cluster-based analysis effectively visualized the extent of the differences between groups even with our relatively small sample sizes. However, a downside of large clusters is that they have low spatial specificity.^49^ The ROI-based analysis, in contrast, facilitated the comparison of different *a priori*-defined brain networks and allowed for the precise localization to specific anatomical regions.

A limitation of this study is that it might not be feasible to disentangle VH and more severe disease course in groups that are matched for age and disease duration, because the presence of VH is a well-known indicator of a more severe disease course.^2^ We found a significantly higher Hoehn and Yahr score in the VH+ group than in the VH− group (although the median score was two for both VH groups), and the comparisons with healthy controls seem to reflect a generally more advanced cholinergic deficiency in the VH+ group than in the VH− group (Supplementary Fig. 1). Nonetheless, the topology of the results of the direct comparison between both VH groups, largely covering the ventral visual stream and parts of the attentional networks, is in strong alignment with the PAD model, suggesting that the differences noted here are specific to the presence of VH.

Another limitation is the lack of data on visual acuity, because this might be an important factor in the development of VH.^50^

## Conclusion

Patients with PD showed a clear global reduction in central cholinergic innervation compared with age- and cognition-matched healthy controls. Moreover, we found a significantly larger decrease in patients suffering from VH compared with those without VH, especially related to the ventral visual stream and the superior temporal lobe, with the largest differences in the left inferior temporal gyrus and the left superior temporal gyrus. Our results confirm the important role of the cholinergic system in the pathology of VH in PD and provide a neurochemical basis for the application of cholinesterase inhibitors in the treatment of VH in Parkinson’s disease.

## Supplementary Material

awae186_Supplementary_Data

## Data Availability

The data of this study can be made available upon request to the corresponding author.
