# Supplementary material for: Cholinergic deficiency in Parkinson’s disease patients with visual hallucinations
Source: Brain. 2024 Jun 12;147(10):3370–8. doi: 10.1093/brain/awae186 (PMC11449127; doi:10.1093/brain/awae186)
Supplement: awae186_Supplementary_Data [file awae186_supplementary_data.pdf]

Inclusion and exclusion criteria

Inclusion

- Willingness to cooperate and sign written informed consent

Patients:

- Clinical diagnosis of idiopathic Parkinson's disease by a neurologist

Exclusion

- Incapable to provide informed consent (e.g. in case of severe dementia)
- Treatment with drugs with anticholinergic activity, as listed in Chew et al. 2008 and Ehrt et al. 2010
- Current or recent treatment with ChEIs
- Presence of deep brain stimulation implants
- (Suspected) pregnancy or active breast feeding
- Migraine
- Epilepsy
- Participation in a scientific research study during the past year involving radiation
- MRI contra-indications, e.g.
    - Ferrous objects in or around the body (e.g. braces, pacemaker, metal fragments)
    - Claustrophobia
- Insufficient knowledge of the Dutch language

Control subjects:

- History of neurological or neurodegenerative disorder

Regions of interest

Supplementary Table 1 Selection of Regions of Interest

Visual	Ventral attentional	Dorsal Attentional	Thalamic
• Superior occipital gyrus	• Superior frontal gyrus	• Angular gyrus	• Lateral geniculate nucleus
• Middle occipital gyrus	• Inferior frontal gyrus, triangular part	• Middle temporal gyrus	• Mediodorsal nucleus
• Inferior occipital gyrus	• Frontal operculum	• Superior parietal lobe	
• Cuneus	• Superior temporal gyrus	• Precentral gyrus	
• Calcarine cortex	• Planum temporale	• Middle frontal gyrus	
• Lungual gyrus		• Precuneus	
• Occipital fusiform gyrus			
• Fusiform gyrus			
• Inferior temporal gyrus			

Cluster-based analysis

Visual hallucinations and no visual hallucinations versus healthy controls

In the PD subgroup without VH, we found a cluster (41,541 voxels) of lower tracer uptake compared to healthy controls, primarily in the occipital lobe, but extending towards both left

and right parietal and temporal lobes (Supplementary Figure 1A). A separate, smaller cluster (610 voxels) was found in the left precentral and middle frontal gyrus. The largest cluster (98,609 voxels) was found in the PD subgroup with VH, compared to healthy controls (Supplementary Figure 1B). Compared to the VH- cluster, this pattern extended more towards anterior and superior parts of the temporal lobe, especially in the left hemisphere, and also included medial frontal areas. A second, smaller cluster (384 voxels) was found in the left superior frontal gyrus and supplementary motor cortex.

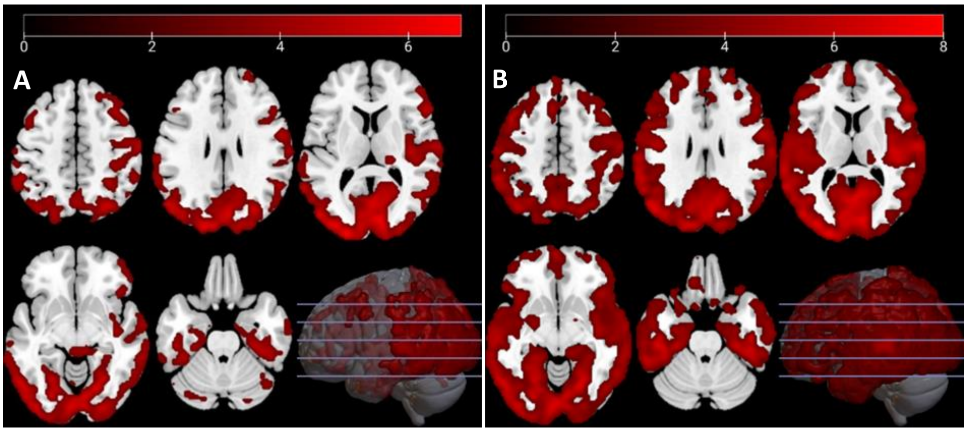

Supplementary Figure 1. Decreased [^{18}F]FEOBV tracer uptake in (A) patients without visual hallucinations (n=20) and (B) with visual hallucinations (n=13) compared to healthy controls (n=10). Depicted are t-values, corrected for age, sex and disease duration.

Post-hoc analysis

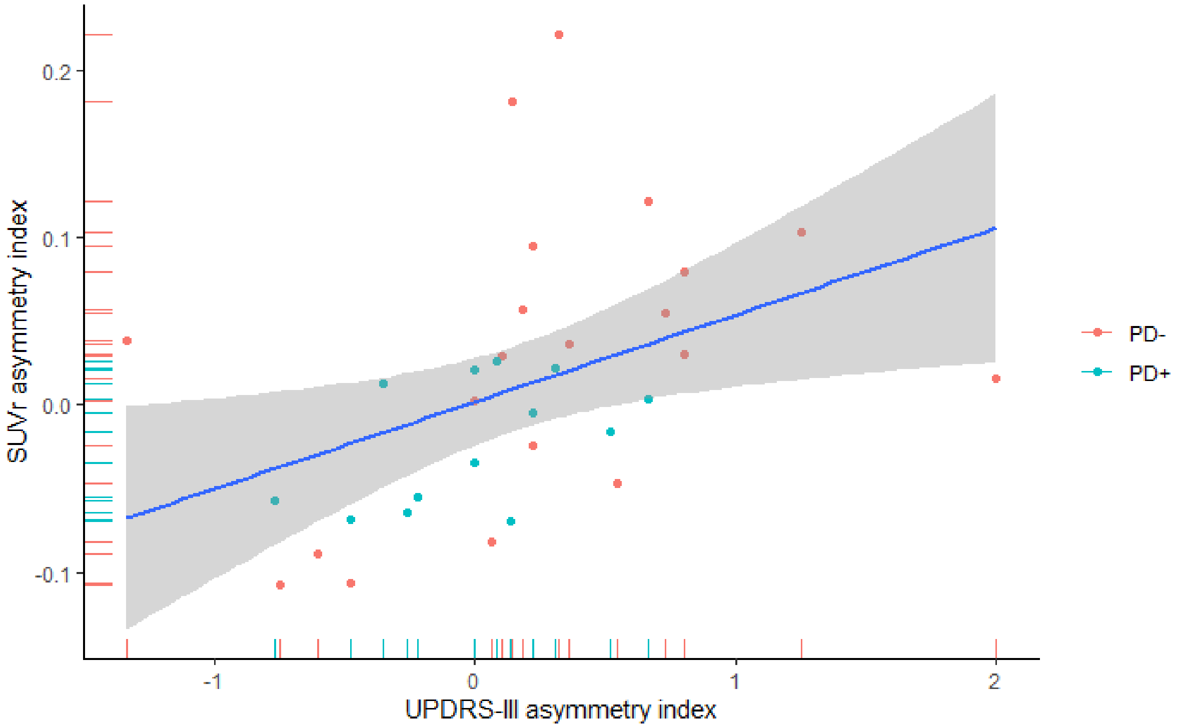

Supplementary Figure 2. Significant correlation between the asymmetry index of tracer uptake in the inferior temporal gyrus and the asymmetry index of the Movemen Disorder Society Unified Parkinson's Disease Rating Scale part 3 (MDS-UPDRS-III) (r=0.42, P=.015). A positive SUVr asymmetry index means a lower tracer uptake in the right hemisphere compared to the left hemisphere and a positive MDS-UPDRS-III asymmetry index means worse motor symptoms in the left extremities compared to the right extremities.
